# Crystal Structure of C-Terminal Coiled-Coil Domain of SYCP1 Reveals Non-Canonical Anti-Parallel Dimeric Structure of Transverse Filament at the Synaptonemal Complex

**DOI:** 10.1371/journal.pone.0161379

**Published:** 2016-08-22

**Authors:** Eun Kyung Seo, Jae Young Choi, Jae-Hee Jeong, Yeon-Gil Kim, Hyun Ho Park

**Affiliations:** 1 School of Chemistry and Biochemistry and Graduate School of Biochemistry at Yeungnam University, Gyeongsan, South Korea; 2 Pohang Accelerator Laboratory, Pohang University of Science and Technology, Pohang, Kyungbuk, South Korea; University of Liverpool, UNITED KINGDOM

## Abstract

The synaptonemal complex protein 1 (SYCP1) is the main structural element of transverse filaments (TFs) of the synaptonemal complex (SC), which is a meiosis-specific complex structure formed at the synapse of homologue chromosomes to hold them together. The N-terminal domain of SYCP1 is known to be located within the central elements (CEs), whereas the C-terminal domain is located toward lateral elements (LEs). SYCP1 is a well-known meiosis marker that is also known to be a prognostic marker in the early stage of several cancers including breast, gliomas, and ovarian cancers. The structure of SC, especially the TF structure formed mainly by SYCP1, remains unclear without any structural information. To elucidate a molecular basis of SC formation and function, we first solved the crystal structure of C-terminal coiled-coil domain of SYCP1. The coiled-coil domain of SYCP1 forms asymmetric, anti-parallel dimers in solution.

## Introduction

Meiotic recombination is essential for genetic exchange, producing healthy haploid gametes for diploid organisms [1]. During this process, homologous chromosomes are aligned next to each other for paring events. These events are mediated by complicated molecular structures known as synaptonemal complexes (SCs), which act as molecular zippers to hold in close apposition pairs of the homologous chromosome [2–5]. The failure of proper assembly of the SC has been shown to be associated with infertility or non-lethal aneuploidy such as Down’s syndrome [6–9].

**Fig 1:**
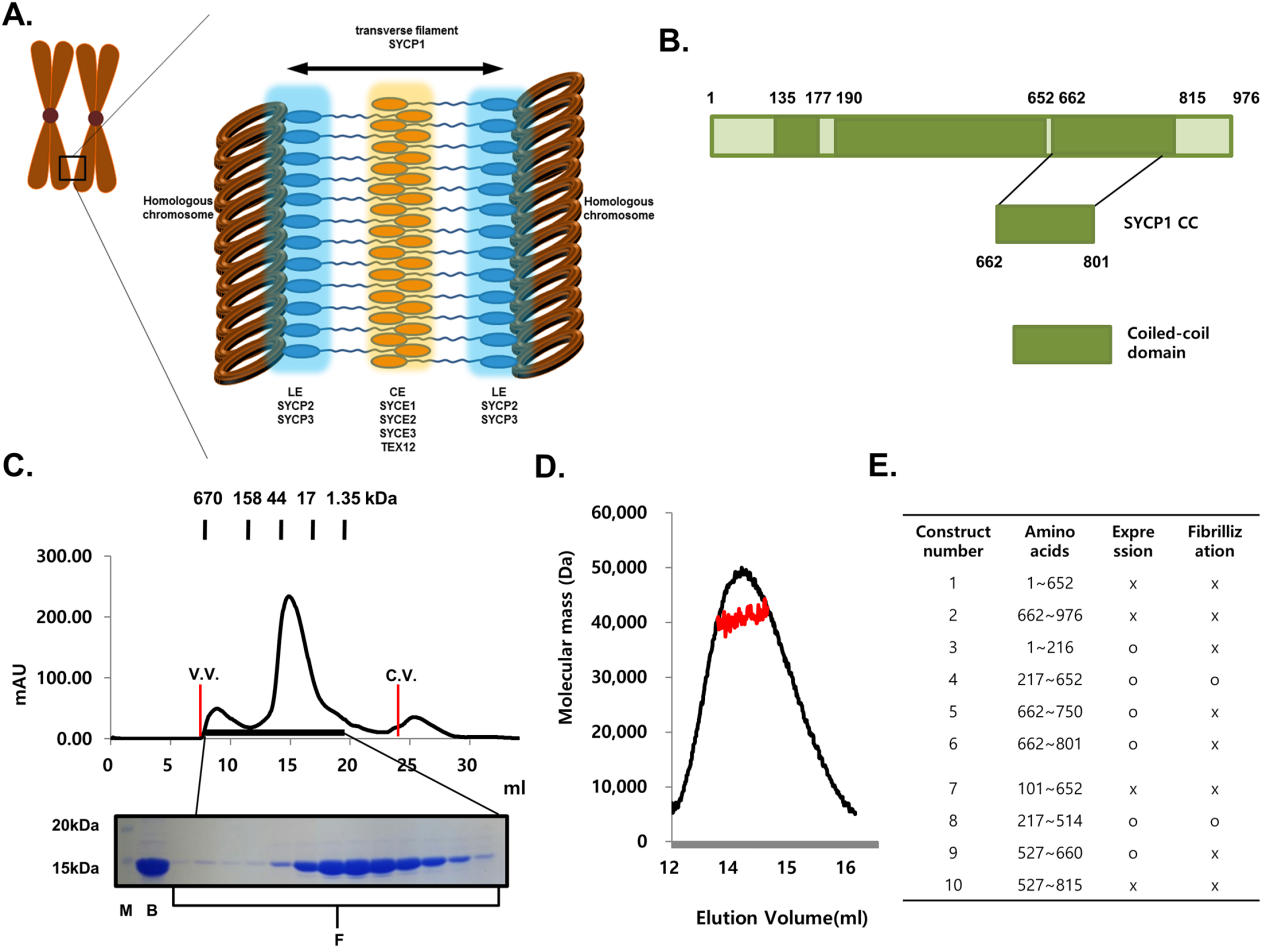
Organization of syneptonemal complex and schematic view of domain organization of SYCP1. (A) The cartoon of the structure of syneptonemal complex. (B) Schematic view of the domain boundary of SYCP1. The construct used for this structural study is magnified. (C) Size-exclusion chromatography profile. SDS-PAGE loaded fractions (black-bar) of Size-exclusionchromatography are shown under the profile. V.V.: Void volume, C.V.: Column volume, M: Marker, B:Sample just before loaded onto size-exclusion chromatography, F: Fractions loaded onto SDS-PAGE (D) Multi-angle light scattering (MALS) results. The red line indicates the experimental molecular weight. (E) Constructs that have been used for expression and purification of SYCP1.

Assembly of the SC is initiated in the early stage of meiotic prophase I, which is commonly divided into five substages, leptotene, zygotene, pachytene, diplotene, and diakinesis [10]. For proper assembly of the syneptonemal complex followed by correct pairing of the homologous chromosome, lateral elements (LEs), which are composed of SYCP2 and SYCP3, should be formed along each chromosome at the initial stage during leptotene. Later, the two LEs associate with the linker part, known as transverse filaments (TFs), which are primarily composed of SYCP1. The central element (CE), which is composed of SYCE1, SYCE2, SYCE3 and TEX12, then connects to the LEs via TFs [3,10,11] (Fig 1A). Electron microscopy has revealed that SC adopts the conserved tripartite structure in all diploid organisms in which it is found, from yeast to humans. Furthermore, the length of TF and CE has been shown to be ~100 nm, whereas LEs and CEs have lengths of ~50 and 40 nm, respectively [12].

All seven essential component proteins in the SC contain predicted coiled-coil domains. The main structural constituent of TFs, SYCP1, is known to contain a large central region of putative coiled-coil domain with flanking N- and C-terminal domains [13] and to form multi-stranded, cross-striated fibers in vivo [14]. It is well known that the N-terminal domain is located within the CE, whereas the C-terminal domain is located next to the LE [15]. Overexpression of SYCP1 produces a synaptonemal complex-like structure, and targeted mutation of this gene in mice results in failure of synapse formation and infertility, indicating that SYCP1 is the main structural element in the SC [16,17]. SYCP1 is a well-known meiosis marker known as a prognostic marker of the early stage of several cancers, including breast, gliomas, and ovarian cancers [18]. Despite the importance of this complex, there is no structural information regarding SC at atomic level, except for the recently elucidated crystal structure of SYCP3 [19]. SYCP3 is one of the main protein components of LEs and formed along each chromosome at the initial stage during meiotic recombination. The structure of SYCP3 was composed of coiled-coil structure. The TF structure formed mainly by SYCP1 remains unclear, without any structural information. To elucidate a molecular basis for the formation and function of SC, we attempted to solve the structure of SYCP1. Here, we provide the first structural information of the central coiled-coil domain of SYCP1 (hereafter known as SYCP1 CC), which is an essential component of TFs. We showed that SYCP1 forms asymmetric, anti-parallel dimers in solution. This novel structure will provide molecular insight into assembly of the human SC.

## Materials and Methods

### Protein expression and purification

To express the C-terminal histidine-tagged SYCP1 CC, the coiled-coil domain region of SYCP1, which corresponds to residues 662 (D)– 801 (L), was cloned into pET24a. The plasmid was transformed into BL21 E. coli competent cells, and its expression in LB medium was induced by treatment with 0.25 mM isopropyl β-D-thiogalactopyranoside (IPTG) overnight at 293 K when the OD600 reached 0.65. Cells expressing the SYCP1 CC were pelleted by centrifugation, resuspended and lysed by sonication in 25 ml lysis buffer (20 mM Tris pH 7.9, 500 mM NaCl and 25 mM imidazole). The lysate was then centrifuged at 16,000 rpm for 30 min at 277 K, after which the supernatant fractions were applied onto a gravity-flow column (Bio-Rad) packed with Ni-NTA affinity resin (Qiagen). Next, the unbound bacterial proteins were removed from the column using 100 ml of washing buffer (20 mM Tris pH 7.9, 500 mM NaCl and 25 mM imidazole). The C-terminal histidine-tagged SYCP1 CC was eluted from the column using elution buffer (20 mM Tris buffer pH 7.9, 500 mM NaCl and 250 mM imidazole). The collected SYCP1 CC was pulled, concentrated, and applied onto a Superdex 200 gel-filtration column (GE Healthcare) that had been pre-equilibrated with a solution of 20 mM Tris-HCl at pH 8.0 and 150 mM NaCl. The protein eluted at around 14–15 ml upon gel-filtration chromatography was collected and concentrated to 8–9 mg/ml for crystallization.

### Crystallization and data collection

Crystallization was conducted at 20°C by the hanging drop vapor-diffusion method using various screening kits. The final crystals used for the x-ray diffraction study were grown on plates by equilibrating a mixture containing 1 μl of protein solution (8–9 mg/ml protein in 20 mM Tris-HCl at pH 8.0 and 150 mM NaCl) and 1 μl of reservoir solution (2.2 M ammonium chloride and 0.1 M sodium acetate at pH 4.6) against 0.4 ml of reservoir solution. Selenomethionine-substituted SYCP1 CC was produced using a previously established method [20] and crystallized similarly. A 2.0 Å single-wavelength anomalous diffraction (SAD) data set was collected at the selenium peak wavelength at the BL-4A beamline of Pohang Accelerator Laboratory (PAL), Republic of Korea. Data processing and scaling were carried out in the HKL2000 package [21]. A 2.0 Å native data set was also collected.

### Structure determination and refinement

The initial SAD structure solution was obtained through identification of four selenium sites using PHENIX AutoSol, and partial automated building into the density-modified experimental map using PHENIX Autobuild [22]. The structure was extended and completed by iterative manual building in Coot [23] and refined using PHENIX Refine [22]. Molecular structure images were generated using the PyMOL Molecular Graphics System, Version 1.3 Schrödinger, LLC.

### Sequence alignment

Amino acid sequences were analyzed using Clustal W (http://www.ebi.ac.kr/Tools/clustalw2/index.html).

### Multi-angle light scattering (MALS)

The molar mass of the SYCP1 CC was determined by MALS. The target protein was injected onto a Superdex 200 HR 10/30 size exclusion column (GE Healthcare) that had been equilibrated in appropriate buffer. The chromatography system was coupled to a three-angle light scattering detector (mini-DAWN EOS) and a refractive index detector (Optilab DSP) (Wyatt Technology). Data were collected every 0.5 s at a flow rate of 0.2 ml/min and analyzed using the ASTRA program, which gave the molar mass and mass distribution (polydispersity) of the sample.

### Mutagenesis

Site-directed mutagenesis was conducted using a Quick-change kit (Stratagene) according to the manufacturer’s protocols. Mutagenesis was then confirmed by sequencing. Mutant proteins were prepared using the same method as described above.

### Circular dichroism spectroscopy

The secondary structures were measured by circular dichroism (CD) spectroscopy using a J-715 spectropolarimeter at the Korea Basic Science Institute in South Korea. The spectra were obtained from 200 to 250 nm at 25°C in a 0.1cm pathlength quartz cuvette using a bandwidth of 1.0 nm, a speed of 50 mm/min, and a 5s response time. The protein samples in the buffer containing 20 mM Tris-HCl at pH 8.0 and 150 mM NaCl were diluted to 0.1 mg/ml prior to use. Four scans were accumulated and averaged, after which the α-helical content was calculated from the molar ellipticity at 222 nm.

### Protein Data Bank accession codes

Coordinates and structural factors were deposited in the Protein Data Bank under PDB ID code 4YTO.

## Results

### Crystal structure of the coiled-coil domain of human SYCP1

Syneptonemal complex (SC) is a large structure that acts as a molecular zipper to hold pairs of homologous chromosomes in close apposition. SC can be divided into three structural parts, lateral elements (LEs), transverse filaments (TFs), and central elements (CEs) (Fig 1A). SYCP1, which is the main structural constituent of TFs, is known to contain large central regions of putative coiled-coil domain (Fig 1A and 1B). Despite the importance of SYCP1 in SC, there is currently no structural information available. To understand the SYCP1 mediated TF formation of the SC, we identified stable constructs of human SYCP1 CC that contain amino acids 662–801 (Fig 1B). We then purified the coiled-coil domain of the C-terminal part of SYCP1 using nickel affinity and gel-filtration chromatography. SYCP1 CC eluted at around 14–15 ml, indicating that it might form dimers in solution (Fig 1C). The absolute molecular mass of SYCP1 CC was confirmed by multi-angle light scattering (MALS). The calculated monomeric molecular weight of SYCP CC, including the C-terminal His-tag, was 17,730 Da, and MALS revealed that the experimental molecular weight was 36,030 Da (1.035% fitting error), with a polydispersity of 1.000 (Fig 1D). The results of MALS also support our supposition that SYCP CC exists as dimer in solution. The structural study of SYCP1 has been especially difficult due to the fibrillization problem. Although SYCP1 usually forms fibers in vitro, we were able to obtain over-expressed constructs by designing, constructing and testing many plasmid constructs. Our expression test with various constructs indicated that coiled-coil domain in the middle part of SYCP1 was responsible for the fibrillization (Fig 1E).

**Fig 2:**
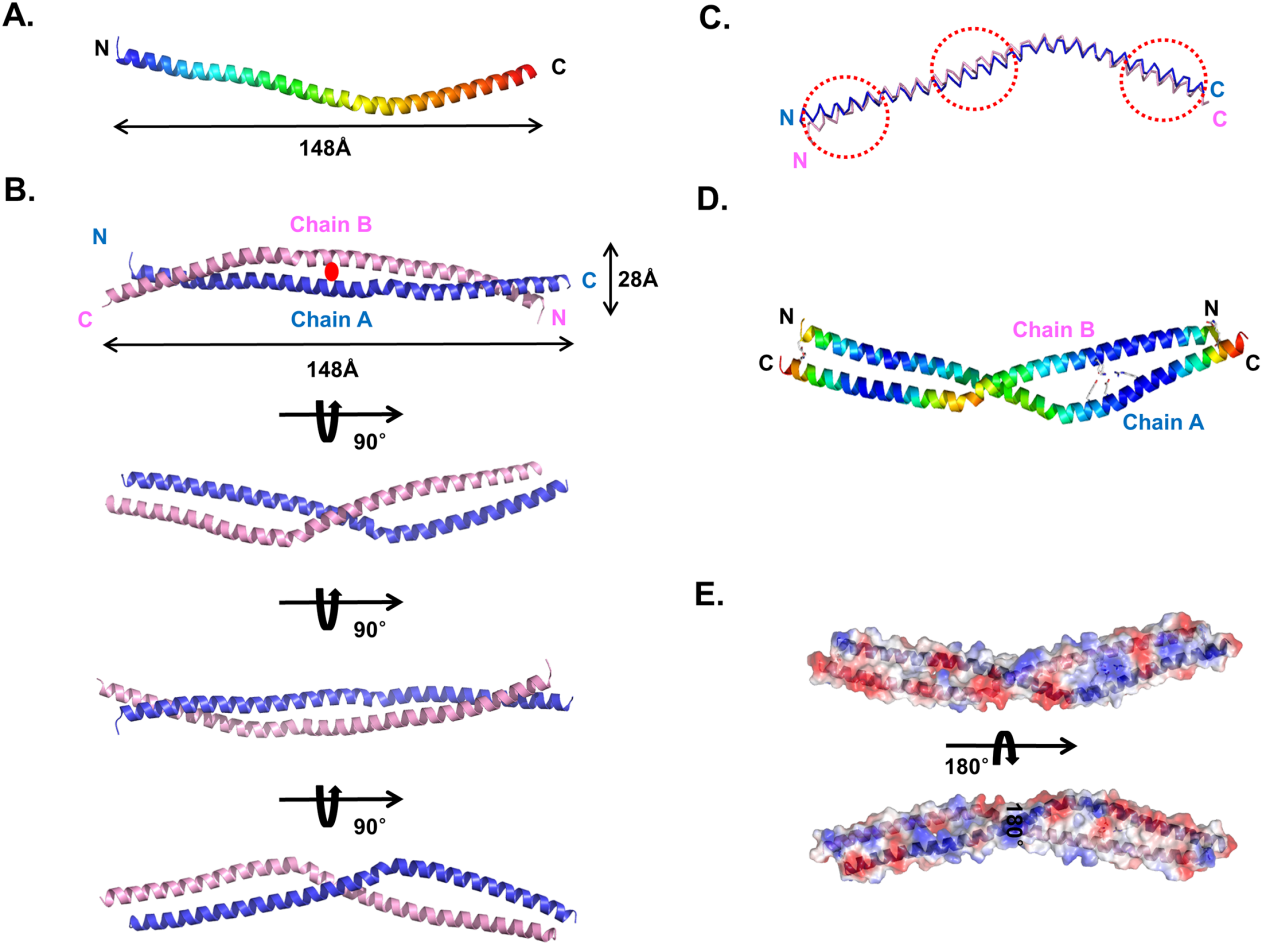
Crystal structure of the SYCP1 CC. (A) Cartoon figure of monomeric SYCP1 CC structure. The chain from the N- to C-termini is colored blue to red. (B) The dimeric structure of the SYCP1 CC detected at the crystallographic asymmetric unit. (C) Superimposition of chain A and chain B. Structurally different regions are indicated by red-dotted squares. (D) B factor distributions are shown by cartoon. Warm and cold colors indicate high and low B-factor levels, respectively. (E) Electrostatic surface representation of SYCP1 CC.

Finally, we solved the 2.0 Å crystal structure using the single-wavelength anomalous diffraction (SAD) method and refined it to an R_work_ = 23.5% and R_free_ = 25.6%. The final atomic model contains residues 673–767 for chain A and 673–769 for chain B (Fig 2A and 2B). The quality of the model was checked using PROCHECK and found to be good, with 100% of the residues are located in the most favorable region of the Ramachandran plot. The data collection and refinement statistics are summarized in Table 1. The high resolution structure of the SYCP1 CC showed that it is an anti-parallel left-handed coiled-coil formed by the two bent helices spanning 148 Å in length and 28 Å in width (Fig 2A and 2B). The two helices wind around each other to form a left-handed coiled-coil. A total dimer is buried at 3600 Å^2^ (a monomer surface area of 1800 Å^2^), which represents ~19% of the total surface area. The equivalent α-carbons of the two helices were superimposed with a root mean square deviation (R.M.S.D.) of 2.3 Å, indicating that chains A and chain B are similar, but not identical (Fig 2C). The average B factor of proteins is 28.1 Å^2^, which indicates the coiled-coil is rigid (Table 1). Plotting individual B factors for each residue showed that the middles of both sides have the lowest B factors, whereas the middle and the end of both sides have the highest B factors, indicating that the bent area is flexible relative to other parts of the helix (Fig 2D). The calculated electrostatic surface showed that the SYCP1 CC surface is a mixture of positively and negatively charged features (Fig 2E) for both sides of the molecules

**Table 1:** Data collection and refinement statistics.

Data collection	Se-Met	Native
Space group	*P4*_*1*_*2*_*1*_*2*	*P4*_*1*_*2*_*1*_*2*
Cell dimensions		
*a*, *b*, *c*	119.60Å, 119.60Å, 84.12Å	118.20Å, 118.20Å, 84.12Å
Resolution	50–2.0 Å	50–2.0 Å
†*R*_sym_	8.1% (45.4%)	9.9% (48.2%)
†*I*/σ(*I*)	30.0 (2.9)	29.5 (3.2)
†Completeness	97.5% (98.9%)	99.4% (98.8%)
†Redundancy	8.4 (7.1)	7.2 (6.1)
**Refinement**		
Resolution	28–2.0 Å	
No. reflections used (completeness)	76,008 (97.4%)	
*R*_work_/*R*_free_	23.3%/25.0%	
No. atoms		
Protein	1549	
Water	155	
Glycerol	12	
Average B-factors		
Protein	28.1 Å^2^	
Water and other small molecules	18.2 Å^2^	
R.M.S. deviations		
Bond lengths	0.007Å	
Bond angles	0.901°	
MolProbity Statistics		
Ramachandran Favored/Outlier (%)	100/0.0	
Rotamer Outliers (%)	0.6	
Clashscore^b^	2.2	
Overall score^b^	1.00	

^†^ Highest resolution shell is shown in parenthesis.

^b^ Scores are ranked according to structures of similar resolution as formulated in MOLPROBITY

### Dimer interface in the coiled-coil domain of SYCP1

**Fig 3:**
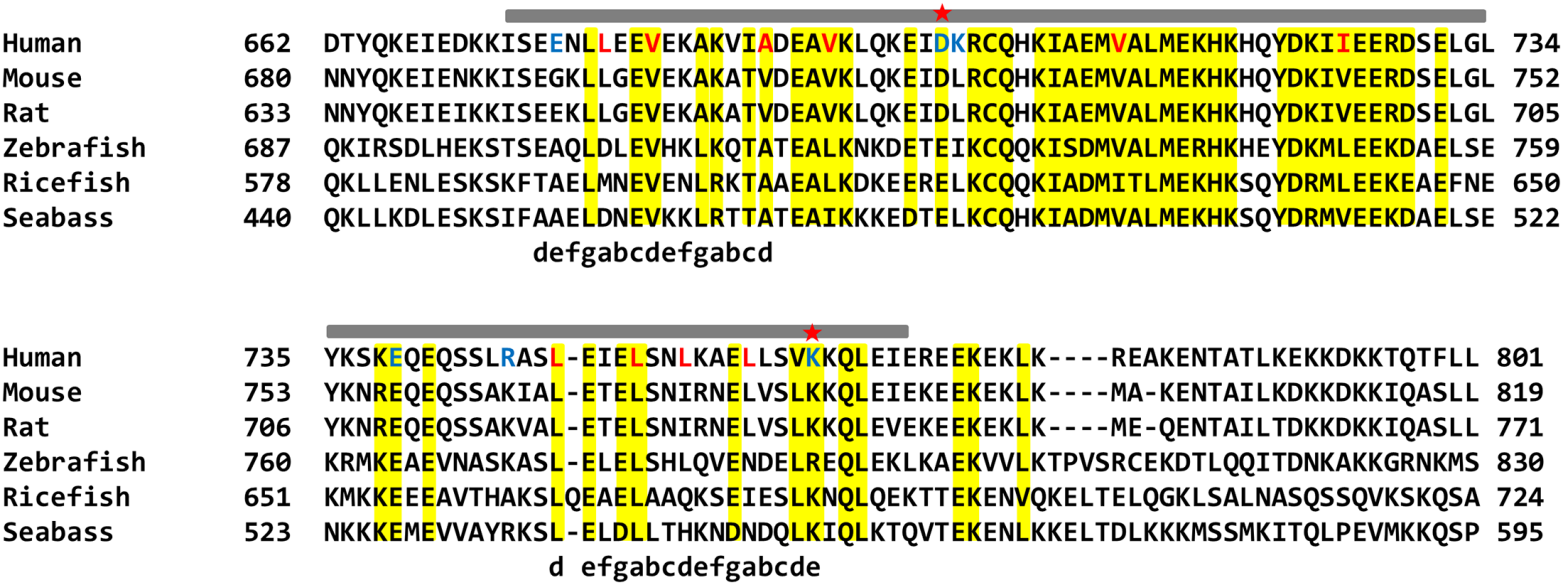
Sequence alignment of SYCP1 CC from different species. The range of solved structures in this study is shown above the sequences. Conserved residues are highlighted with yellow color. Charged and hydrophobic residues that are involved in the interface interaction are shown in blue and red colors, respectively. Red stars indicate mutations that disrupt dimer formations. Heptad repeats are shown below the sequences.

A typical structure of a left-handed coiled-coil exhibits a seven residue heptad repeat, commonly detonated by (abcdefg)_n_. The formation of the coiled-coil structure is mediated by hydrophobic interactions formed by residues at positions a and d and charged-charged interactions formed by residues at positions e and g [24,25]. Heptad repeat in the SYCP1 CC was analyzed by SCOKET server (http://coiledcoils.chm.bris.ac.uk/socket/)[26]. This server could assign heptad repeat only on the beginning and the end of SYCP1 CC structure, indicating that SYCP1 CC is non-canonical anti-parallel coild-coil form (Fig 3A).

**Fig 4:**
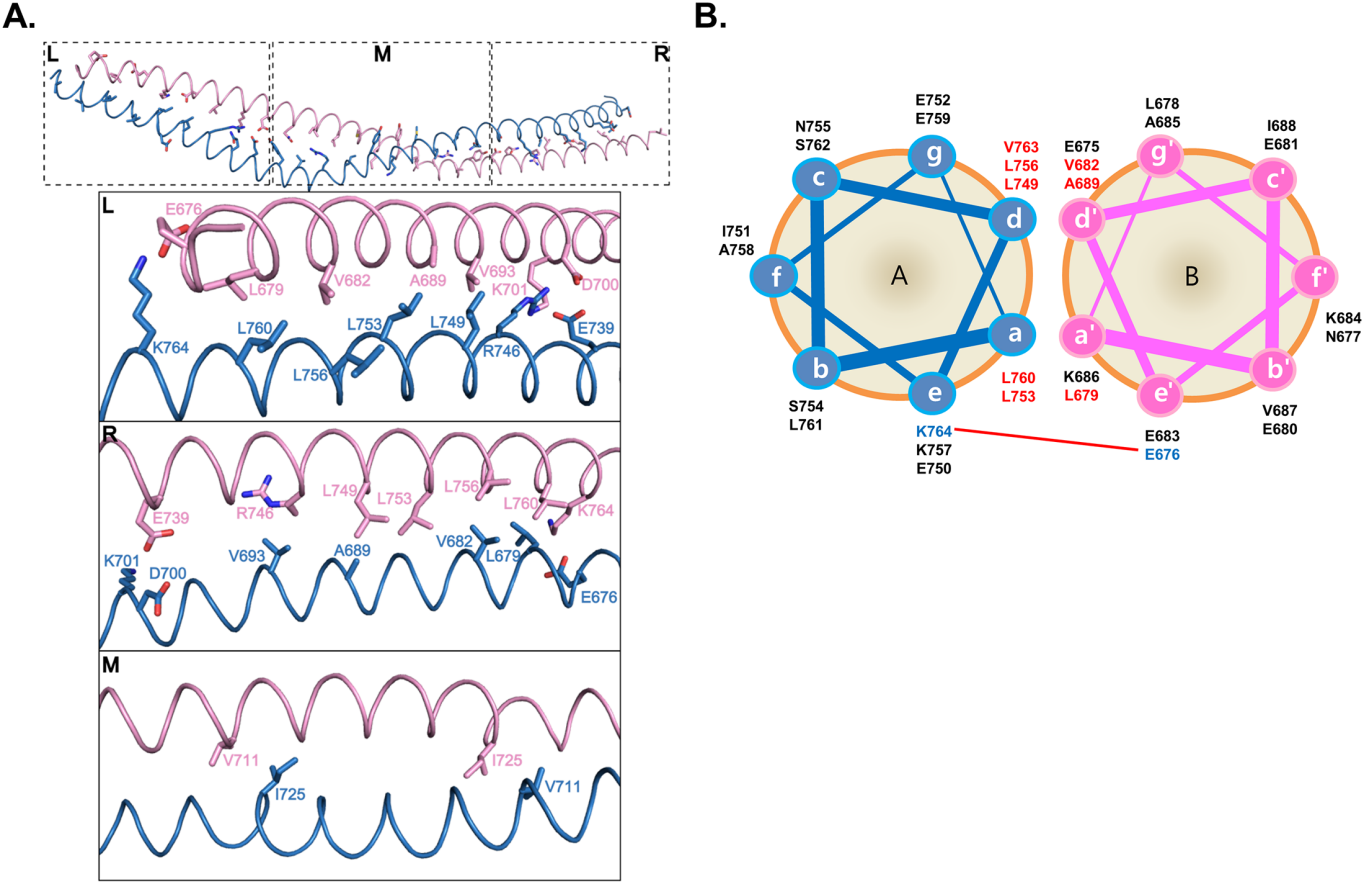
The details of dimeric interfaces of the SYCP1 CC structure. (A) Interhelical interactions of coiled coil within the asymmetric unit of the seleno-methionine crystal are shown. The residues in the positions a and d of heptad repeats are presented as a stick model at the top panel. Close-up views of three different parts are shown. The amino acid residues that participated in the interhelical interactions are labelled. L; Left, M; Middle, R; Right. (B) Helical wheel presentation of the heptad repeats in SYCP1 CC. Interhelical charged interactions are indicated by a red-line connecting paired residues. Charged residues and hydrophobic residues are colored blue and red, respectively.

To enable better visualization of repeated heptad-based hydrophobic and charged interactions in the amino acids sequence of SYCP1 CC, the helical wheel was plotted at Fig 4B. Unlike the other canonical coiled-coil formation (hydrophobic residues primarily located at the a and d positions), only the beginning and the end of the coild-coil were assigned as heptad repeat, occupying hydrophobic residues at a and d positions and charged residues at e and g positions. The highly conserved hydrophobic residues, including L679, A689, V682, L749, L753, L756, L760, and V763, at the a and d positions are involved in stabilizing the antiparallel SYCP1 CC by forming extensive hydrophobic interactions (Fig 4A and 4B). E676 and K764 at e position were participated in the salt bridge formation. D700, K701, E739, and R746 formed charged interaction in the middle part of the coiled-coil structure.

### Charged interactions are critical for stabilization of the coiled-coil structure

**Fig 5:**
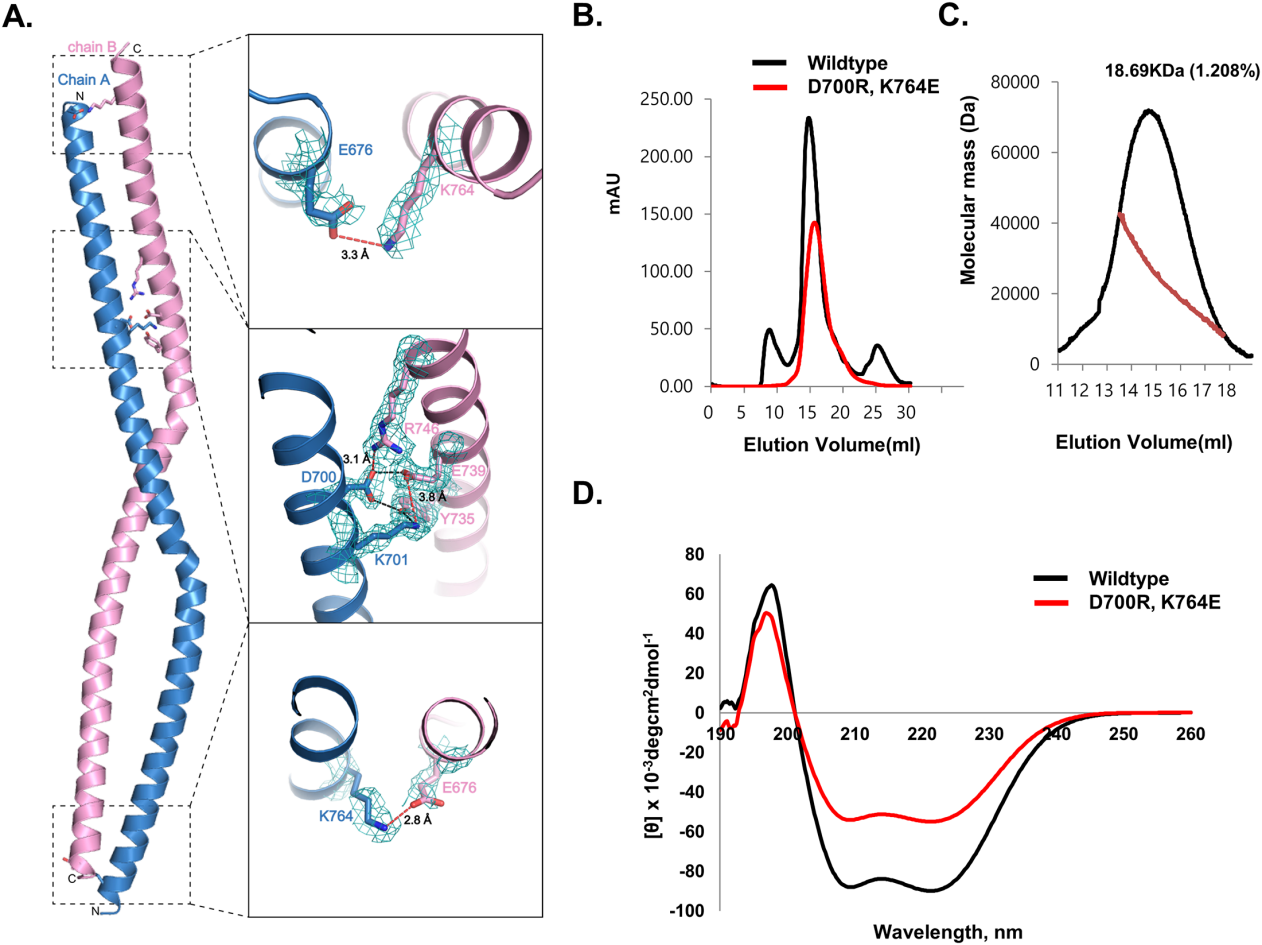
Disruption of the antiparallel left-handed SYCP1 CC. (A) Expected critical residues for formation of the antiparallel left-handed SYCP1 CC based on the structure. The 2Fo-Fc simulated annealing omit map (2.0 Å, 1.2 σ) around the critical residues was calculated with the omission of the model. The red dots lines indicate salt bridges and the black dot lines indicate hydrogen bonds. (B) Gel-filtration profiles of wildtype and double mutant (D700R, K764E). (C) Multi angle light scattering (MALS) with selected peak positions of double mutant (D700R, K764E). The red line indicates the experimental molecular weight. (D) Circular dichroic spectra of wildtype and double mutant (D700R, K764E).

Three charged clusters were detected in the structure, with two located at the end of each side. E676 from one molecule form salt bridges with K764 from another molecule (Fig 5A). The third cluster is formed immediately above the middle part (Fig 5A). D700 and K701 from one molecule form massive salt bridges with E739 and R746 (Fig 5A). The fact that only three charge patches were detected at the asymmetric location of the coiled-coil structure indicated that this coiled-coil structure formed by two molecules is not symmetric and the two molecules are not identical.

Because three charged patches might be critical for stabilization of the coiled-coil structure of SYCP1, we conducted a mutagenesis study to analyze the interface. Two critical residues, D700 and K764, were mutated to opposite charged R (D700R) and E (K764E), respectively, and used for the size-exclusion chromatography and MALS experiments. As shown in Fig 5B, the elution peak of the wildtype observed upon size-exclusion chromatography was moved to a monomeric place by double mutation. The calculated molecular weight of D700R, K764E double mutants from MALS was 18,690 Da (1.1% fitting error), with a polydispersity of 1.000. Poor molecular fitting at MALS experiment might be because of the mixture of monomers and dimers. These findings indicated that the dimeric SYCP1 CC became a monomer via double mutation (Fig 5C). To demonstrate that changes in oligomerization in response to mutation were not by structural disruptions caused by mutation, we evaluated the far UV circular dichroic spectra. As shown in Fig 5D, D700R, K764E double mutant showed CD spectrum patterns typical of α-helical proteins, exhibiting two pronounced minima at 208 nm and 222 nm and a maxima at 195 nm, which is similar to that of the wildtype. Taken together, our mutation study indicates that three charged patches formed by massive salt bridges are critical for stabilization of this anti-parallel left-handed coiled-coil structure of SYCP1.

**Fig 6:**
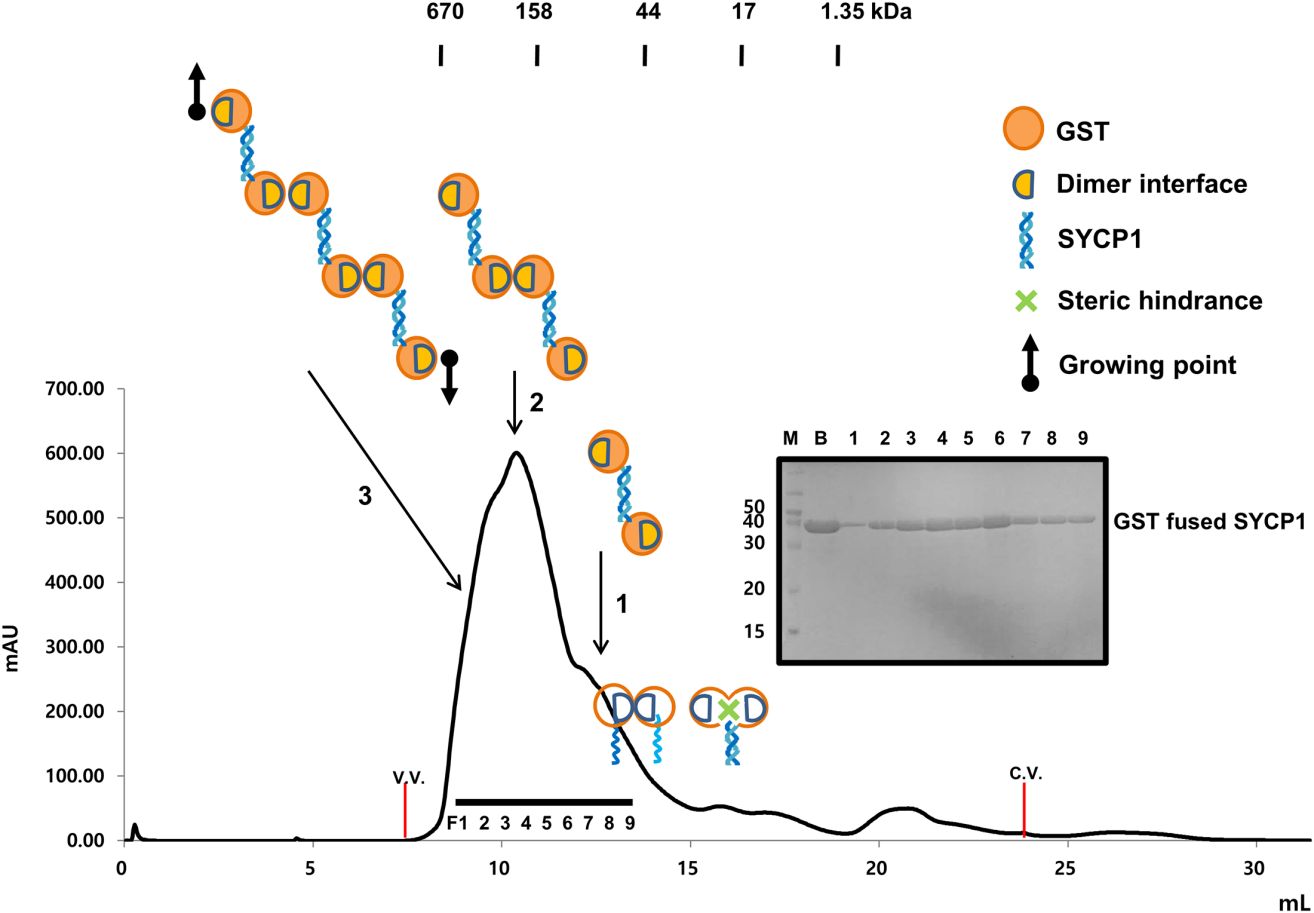
Evidence of antiparallel left-handed SYCP1 CC in solution. Size-exclusion chromatography profile of GST-fused SYCP1 CC. SDS-PAGE loaded fractions (black-bar) of Size-exclusion chromatography are shown next to the profile. V.V.: Void volume, C.V.: Column volume, M: Marker, B:Sample just before loaded onto size-exclusion chromatography, F: Fractions loaded onto SDS-PAGE, 1: Dimer of GST-fused SYCP1 CC position, 2: Tetramer position, 3: higher oligomer position. Cartoons of oligomeric states of GST-fused SYCP1 CC are shown.

To confirm that our structural detection of non-canonical anti-parellel structure is real in solution, we performed steric hindrance assay with GST-fused SYCP1 CC. If SYCP1 CC form parallel coiled-coil, GST-fused SYCP1 CC can exist monomer or dimer in solution. Because GST (which is 28 kDa globular protein) in same location can hinder coiled-coil formation, GST fused SYCP1 CCexist as a monomer. Also we can expect dimer because GST usually form a dimer in solution. Therefore, GST mediated dimerization can be possibly formed. On the other hands, if SYCP1 CC form anti-parallel coiled-coil, which is detected at our crystal structure, GST-fused SYCP1 CC can exist dimer, tetramer, or even higher oligomeric complex. Because GST form stable dimer in solution, anti-parallel GST-fused SYCP1 CC can bind another anti-parallel GST fused-SYCP1 CC in both direction, causing putative tetramer or even higher oligomeric GST-fused SYCP1 (Fig 6). Based on this assumption, we performed size-exclusion chromatography with GST-fused SYCP1 CC. As expected, we can observe dimer, tetramer and even higher oligomer peak in the FPLC profile (Fig 6). This result with other mutation study strongly indicated that our anti-parallel structure of SYCP1 CC is real in solution.

## Discussion

**Fig 7:**
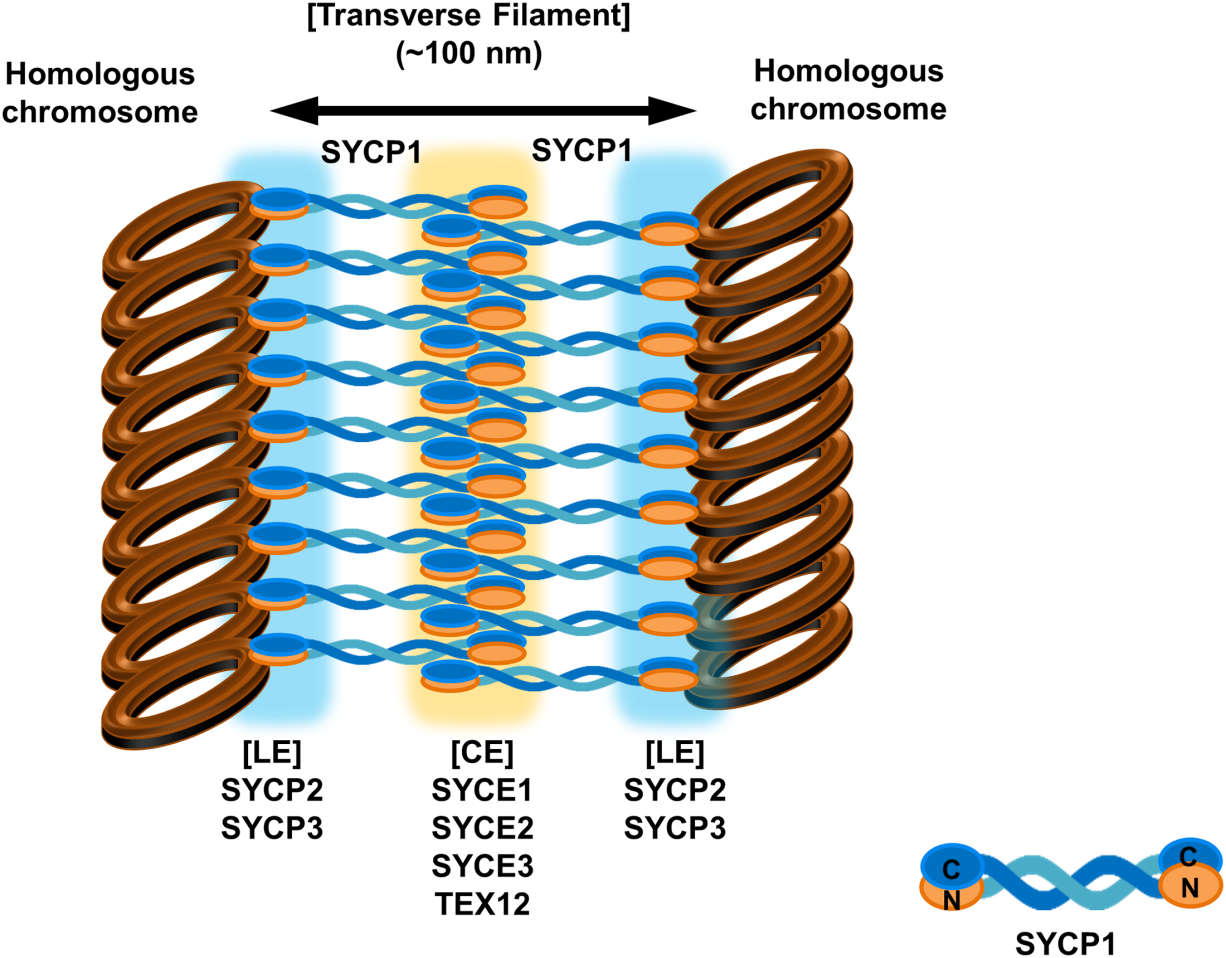
Putative model of synaptonemal complex based on the current structure of non-canonical anti-parallel coild-coil structure of SYCP1.

Since the initial discovery of the synaptonemal complex in 1956 as a physical hallmark of meiosis, the molecular structure and assembly mechanism of the tripartite structure of the synaptonemal complex have remained largely unknown. This is because it has been difficult to produce recombinant proteins composed of synaptonemal complex for use in structural and biochemical studies. However, structural and biochemical studies of several synaptonemal complex components including SYCE2-TEX12 complex and SYCP3 have been recently reported as the production of recombinant proteins has improved [19,27]. The crystal structure of SYCP3, which is the first crystal structure of the component protein of synaptonemal complex, was recently solved and the results showed that SYCP3-DNA mediated assembly of lateral element (LE) of the synaptonemal complex [19]. However, the linker part of synaptonemal complex known as transverse filaments (TFs), which is mainly formed by SYCP1, is still not clear. Previous electron microscopy studies of the native SC showed that SYCP1 N-termini are found in the central elements and C-termini in the lateral elements, indicating that SYCP1 molecules exist as parallel coiled-coils [15,28]. Current structural study of the C-terminal coiled-coil domain of SYCP1 showed that the TF structure formed by SYCP1 might be an anti-parallel left-handed coiled-coil structure. This anti-parallel left-handed coiled-coil structure of SYCP1 might be the basic unit of TFs, and one N-terminal and C-terminal part of SYCP1 can be located on each side of the LEs (Fig 7). Our structure is controversial to the previously known fact that the N-terminal domain of SYCP1 is located within the central elements (CEs), whereas the C-terminal domain is located toward lateral elements (LEs). Because the construct studied is just 140 amino acids (forming a 15 nm long coiled-coil) and is insufficient to span the full 50 nm distance between central and lateral elements, our current structural study has limitation to predict full-length SYCP1 structure and transverse filaments in SC. However, our current crystallographic study will present another possible structure of transverse filaments (TFs) in syneptonemal complex. Non-canonical coiled-coil structures have been detected in the several important molecules that are especially involved in the membrane trafficking [29,30]. Since imperfect non-canonical coiled coil structure of Beclin1 is critical for the homodimer and heterodimer formation with its binding partner, imperfect non-canonical coiled coil structure of SYCP1 detected in this structure might be also important for the homodimerization and heterodimerization with its binding partner for the proper function of SYCP1 in the synaptonemal complex. Loosely packed homo-dimeric coiled-coil structure would have a benefit for exchanging its binding partner when necessary.
